# Impact of sarcopenia and sarcopenic obesity on survival in patients with primary liver cancer: a systematic review and meta-analysis

**DOI:** 10.3389/fnut.2023.1233973

**Published:** 2023-10-19

**Authors:** Xuanmei Li, Xue Huang, Lifu Lei, Shiwen Tong

**Affiliations:** ^1^Department of Infectious Diseases, Department of Clinical Nutrition, Key Laboratory of Molecular Biology for Infectious Diseases (Ministry of Education), Institute for Viral Hepatitis, The Second Affiliated Hospital, Chongqing Medical University, Chongqing, China; ^2^Department of Ultrasound, The Second Affiliated Hospital, Chongqing Medical University, Chongqing, China; ^3^Department of Clinical Nutrition, The Second Affiliated Hospital, Chongqing Medical University, Chongqing, China

**Keywords:** primary liver cancer, sarcopenia, sarcopenic obesity, muscle depletion, survival

## Abstract

**Background:**

Sarcopenia and sarcopenic obesity are associated with an increased possibility of adverse clinical outcomes; however, the effects of sarcopenia and sarcopenic obesity on patients with primary liver cancer remain controversial. Therefore, the present study aimed to determine the impact of sarcopenia and sarcopenic obesity on survival in patients with primary liver cancer.

**Methods:**

We searched studies published in English in PubMed, Embase, Web of Science, and Cochrane Library databases up to 13 November 2022. Cohort studies that reported the association among sarcopenia, sarcopenic obesity, and patient survival were included.

**Results:**

A total of 64 cohort studies with data on 11,970 patients with primary liver cancer were included in the meta-analysis. Sarcopenia was associated with poor overall survival in patients with primary liver cancer [adjusted hazard ratio (HR) 2.11, 95% confidence interval (CI): 1.89–2.36, *P* < 0.0001], with similar findings for sarcopenic obesity (adjusted HR: 2.87, 95% CI: 2.23–3.70, *P* < 0.0001). Sarcopenia was also associated with poor overall survival across the subgroups analyzed by ethnicity, type of liver cancer, treatment modalities, method used to define sarcopenia, and etiology of liver cancer. We also found a negative correlation among sarcopenia, sarcopenic obesity, and recurrence-free/disease-free survival (adjusted HR: 1.73, 95% CI: 1.50–1.99, *P* < 0.001; adjusted HR: 2.28, 95% CI: 1.54–3.35, *P* < 0.001, respectively).

**Conclusion:**

Sarcopenia and sarcopenic obesity were significantly associated with poor overall survival and recurrence-free/disease-free survival in patients with primary liver cancer.

**Systematic review registration:**

https://www.crd.york.ac.uk/prospero/display_record.php?RecordID=378433, PROSPERO [42022378433].

## 1. Introduction

Primary liver cancer is the sixth most frequently occurring cancer and ranks as the third leading cause of cancer-related mortality worldwide, accounting for 8.3% of total cancer deaths (1), thus resulting in a global medical and economic burden. Hepatocellular carcinoma (HCC) is the predominant type of primary liver cancer, comprising 75%−85% of the cases, and intrahepatic cholangiocarcinoma (ICC) follows as the subsequent type (1). There are significant gender and racial differences in morbidity and mortality due to primary liver cancer; both morbidity and mortality rates are two-fold to three-fold higher in men than in women in most regions, and the disease is more common among Asians due to a high prevalence of hepatitis B and C (1). It is therefore critical to identify patients with high mortality risk based on the patient's prognosis for determining individualized treatments and improving the survival rate of patients with primary liver cancer. In recent years, researchers have made several efforts to determine the factors that influence the clinical outcomes of patients with liver cancer. Thus far, the Barcelona Clinic Liver Cancer (BCLC), Model for End-Stage Liver Disease (MELD), and the albumin–bilirubin (ALBI) scores have been used clinically to evaluate the prognosis of patients with liver cancer; however, these prognostic tools cannot adequately capture the nutritional and functional status of these patients.

Sarcopenia, as a marker of malnutrition, has been defined by the European Working Group on Sarcopenia in Older People (EWGSOP2) in 2018 as the presence of both low muscle mass and low muscle function (strength or performance) (2). It is difficult to diagnose sarcopenia because of different measuring methods and cutoff values. Sarcopenia is usually evaluated based on grip strength, dual-energy X-ray absorptiometry, computed tomography (CT), and magnetic resonance imaging (MRI) (2). Sarcopenia increases the risk of worse clinical outcomes such as reduced quality of life, development of complications, higher hospitalization cost, and death (3–6). Previous studies have shown that the hospitalization cost of older patients with sarcopenia on admission was five-fold more than those without sarcopenia (7). Sarcopenia is a common condition in patients with oncological and chronic diseases. In a systematic review and meta-analysis that included 38 studies on sarcopenia and solid cancer outcomes, sarcopenia was significantly associated with the poor overall survival of patients (8). Similarly, according to a recent umbrella review of meta-analyses, sarcopenia is associated with adverse clinical outcomes across 12 cancer types: gastric, hepatocellular, urothelial, head and neck, hematologic malignancy, pancreatic, breast, colorectal, lung, esophageal, hematologic malignancies, and ovarian (9). The existence of sarcopenia was found to be associated with a higher risk of death in patients with liver cirrhosis (10), which is likely to progress into liver cancer.

The rate of fat deposition tends to increase in sarcopenic patients, resulting in systemic inflammatory activation and insulin resistance, which subsequently leads to progressive muscle reduction and fat accumulation, especially in conditions such as aging and cachexia (11). This vicious cycle finally results in sarcopenic obesity (SO), which is defined as the co-existence of obesity and sarcopenia (12). More recently, SO has received increasing interest from oncologists because of its adverse outcomes in patients with cancer. SO is an independent prognostic factor affecting the risk of adverse outcomes in oncological patients (13–15).

Several studies, however, reported no association between sarcopenia and prognosis in patients with HCC (16–20). The influence of sarcopenia on survival in patients with liver cancer remains controversial, and a comprehensive analysis based on evidence-based medicine is required. To the best of our knowledge, previous meta-analyses have focused only on HCC patients and did not include data on patients with ICC. Recently, an increasing number of studies have examined the prognostic factors of liver cancer patients. Hence, we analyzed and summarized the relationship among sarcopenia, SO, and survival in patients with primary liver cancer. Our study aimed to determine the impact of sarcopenia and SO on the survival of patients with primary liver cancer.

## 2. Methods

### 2.1. Search strategy

We searched studies relevant to the association of sarcopenia, SO, and survival of patients with liver cancer in PubMed, Embase, Web of Science, and Cochrane Library databases up to 13 November 2022. The search keywords included sarcopenia, muscle depletion, muscle weakness, liver cancer, and liver neoplasm. The detailed search strategies are presented in Supplementary Table S1. We restricted the studies to those published in English and conducted on humans. We also retrieved potential studies by reading through the relevant systematic reviews and meta-analyses. The present meta-analysis adhered to the PRISMA guidelines (21), and its protocol was registered on PROSPERO (CRD 42022378433).

### 2.2. Criteria for selection

Studies that met the following criteria were included: (1) participants: patients with liver cancer confirmed by clinical/imaging or liver biopsy criteria (may include patients evaluated or already listed for liver transplantation), including HCC and ICC; (2) exposures: pretreatment for sarcopenia and/or SO; (3) outcomes: the impact of sarcopenia and/or SO on patient survival; and (4) study design: prospective or retrospective cohort study.

Studies that met the following criteria were excluded: (1) studies lacking the criteria for diagnosing sarcopenia or SO; (2) studies lacking the statistical data on the impact of sarcopenia and/or SO on survival [hazard ratio (HR) and 95% confidence interval (CI)]; and (3) reviews, case reports, editorials, letters, posters, and/or conference abstracts.

The authors XL and XH independently screened the title/abstract of all the identified citations for eligibility by using the abovementioned inclusion/exclusion criteria. Next, they retrieved and rescreened the full texts of relevant articles. For studies with overlapping cohorts, studies having the latest data and/or a larger sample size and/or more data available for subgroup analysis were used. Disagreements were resolved by consensus or discussion.

### 2.3. Data acquisition and quality assessment

XL and XH extracted the following data independently from each included study: first author's name, first author's country, published year, ethnicity, study type, type of liver cancer, treatment modalities, etiology of liver cancer, enrolled numbers, patient demographics (including age and sex), duration of follow-up, definitions of sarcopenia and SO, cutoff values of sarcopenia, and number of sarcopenia or SO patients. The quality of the enrolled studies was independently evaluated by the two authors according to the Newcastle–Ottawa Scale (NOS). Discrepancies between both investigators were resolved by consensus and discussion.

### 2.4. Statistical analysis

The outcomes for the association between sarcopenia and overall survival (OS), recurrence-free survival (RFS), or disease-free survival (DFS) were reported as crude and adjusted HR with corresponding 95% CI values. HR and 95% CI values were directly extracted from univariate and multivariate Cox regression analyses. The impact of sarcopenia and SO on the OS of primary liver cancer patients was assessed by the pooled unadjusted HR or adjusted HR and 95% CI by using a random-effects model (DerSimonian–Laird method) (22). A subgroup analysis for the adjusted HRs was conducted according to ethnicity, type of liver cancer, treatment modalities, method used to define sarcopenia, and etiology of liver cancer. Cochran's *Q* statistic and *I*^2^ were used to evaluate heterogeneity, with a *P*-value of <0.1 and *I*^2^ > 50% considered to show meaningful heterogeneity (23). We assessed publication bias through the utilization of funnel plots in meta-analysis and quantified it using Egger's regression test. We also conducted a meta-analysis of single proportions to determine the prevalence of sarcopenia. All analyses were performed with STATA software v15.0 (StataCorp, College Station, TX, USA), and a *P*-value of <0.05 was considered to be statistically significant.

## 3. Results

### 3.1. Study search and characteristics

**Figure 1:**
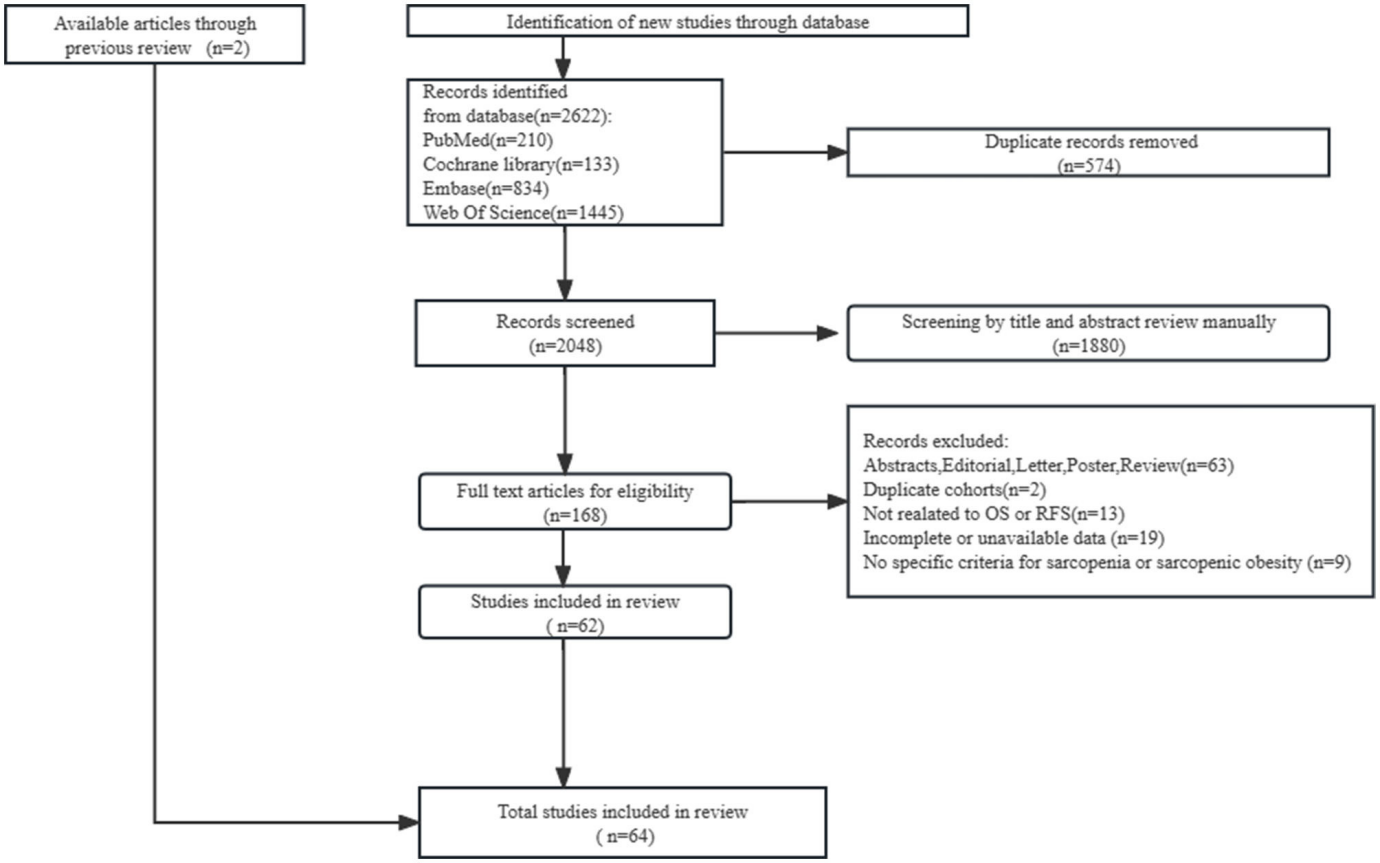
PRISMA flow diagram of the search strategy.

The search in PubMed, Embase, Web of Science, and Cochrane Library databases yielded 2,622 relevant citations, of which 574 duplicates and 1,880 unavailable titles/abstracts were excluded. After reviewing the full text of the remaining 168 publications and previous reviews, we included 64 eligible cohort studies with 11,970 patients (Figure 1).

The demographics and characteristics of the included studies are shown in Table 1. In general, 47 of the 64 included studies were conducted in Asia, predominantly in Japan (*n* = 31), and 17 studies were from non-Asian regions. Four studies were prospective, and 60 studies were retrospective.

**Table 1:** Summary characteristics of the included studies.

**References**	**Country**	**Published year**	**Ethnicity**	**Study type**	**Type of liver cancer**	**Treatment modalities**	**Etiology of liver cancer and number**	**Enrolled number (male/ female)**	**Age in years^a^**	**Follow-up^a^**	**Sarcopenia definition**	**Cut-off value (male/ female, cm/m^2^)**	**No. of sarcopenia (male/ female)**
Meza-Junco et al. (24)	Canada	2013	Caucasian	Prospective	HCC	Liver transplant	Alcohol 13, HBV 16, HCV 53, alcohol + HCV 23, NASH 8, others 3	116 (98/18)	58^b^	12 months	L3-SMI	53 (BMI ≥ 25) or 43 (BMI < 25)/41	35 (30/5)
Itoh et al. (25)	Japan	2014	Asian	Retrospective	HCC	Hepatectomy	NR	190 (146/44)	68 (low visceral area); 69 (high visceral area)^b^	NR	L3-SMI	43.75/41.10	77 (NR)
Fujiwara et al. (26)	Japan	2015	Asian	Retrospective	HCC	Different treatments	HBV 142, HCV 895, HCV + HBV 13, none 207	1,257 (828/429)	68.8^b^	SEVEN	L3-SMI	36.2/29.6	139 (96/43)
Harimoto et al. (27)	Japan	2015	Asian	Retrospective	HCC	Hepatectomy	HBV 8, HCV 51	139 (98/41)	76.5 (sarcopenia); 75.9 (non-sarcopenia)^b^	NR	L3-SMI	43·75/41·10	57 (40/17)
Iritani et al. (28)	Japan	2015	Asian	Retrospective	HCC	Different treatments	HBV 28, HCV 134, HBV + HCV 3, others 52	217 (146/71)	72	637 days	L3-SMI	1.24	24 (NR)
Levolger et al. (29)	Netherlands	2015	Caucasian	Retrospective	HCC	Hepatectomy or RFA	HBV 15, HCV 22	90 (63/27)	62	22.5 months	L3-SMI	52/39.5	52 (39/13)
Valero et al. (16)	USA	2015	Mixed	Retrospective	Primary liver cancer	Hepatectomy or OLT	HBV 10, HCV 28, HBV + HCV 2, none 56	96 (59/37)	61.9^b^	NR	L3-TPA	784.0/642.1 mm^2^/m^2^	47 (NR)
Voron et al. (30)	France	2015	Caucasian	Retrospective	HCC	Hepatectomy	Alcohol 12, HBV 22, HCV 27, NASH 11, multifactorial 8 l, unknown 29	109 (92/17)	61.66^b^	21.23 months	L3-SMI	52.4/38.9	59 (53/6)
Zhou et al. (31)	China	2015	Asian	Retrospective	ICC	Hepatectomy	NR	67 (22/45)	61	NR	L3-SMI	43.75/41.10	33 (9/24)
Higashi et al. (32)	Japan	2016	Asian	Retrospective	HCC	Hepatectomy	HBV 28, HCV 43, NBNC 101	144 (108/36)	65.1^b^	NR	L3-SMI	43.2/35.3	72 (54/18)
Itoh et al. (33)	Japan	2016	Asian	Retrospective	HCC	LDLT	HCV 110	153 (86/67)	57 (low-SVR); 58 (not low-SVR)	5.2 years	None	None	None
Kamachi et al. (34)	Japan	2016	Asian	Retrospective	HCC	Hepatectomy or RFA	HCV 92	92 (65/27)	73 (sarcopenia); 70 (non-sarcopenia)	34.0 months	L3-SMI	52.4/38.5	61 (51/10)
Takagi et al. (35)	Japan	2016	Asian	Retrospective	HCC	Hepatectomy	HBV and/or HCV 171, others 83	254 (207/47)	65.7^b^	NR	L3-SMI	46.4/37.6	118 (93/25)
Yabusaki et al. (36)	Japan	2016	Asian	Retrospective	HCC	Hepatectomy	HBV 50, HCV 88, HBV+HCV 1, others 56	195 (157/38)	66^b^	1,121 days	L3-SMI	43.75/41.10	89 (57/32)
Begini et al. (37)	Italy	2017	Caucasian	Retrospective	HCC	Different treatments	HBV 11, HCV 40, alcohol 22, NASH 19	92 (65/27)	71.60	NR	L3-SMI	53 (BMI ≥ 25) or 43 (BMI < 25)/41	37 (20/17)
Hiraoka et al. (38)	Japan	2017	Asian	Retrospective	HCC	Sorafenib	HBV 18, HCV 56, HBV + HCV 2, NBNC 17	93 (81/12)	68.8 (sarcopenia); 68.2 (non-sarcopenia)^b^	NR	L3-PMI	4.24/2.50	20 (19/1)
Nishikawa et al. (39)	Japan	2017	Asian	Retrospective	HCC	Sorafenib	HBV 33, HCV 144, HBV + HCV 4, NBNC 49, unknown 2	232 (181/51)	72	NR	L3-SMI	36.2/29.6	151 (126/25)
Okumura et al. (40)	Japan	2017	Asian	Retrospective	ICC	Hepatectomy	NR	109 (67/42)	68	NR	L3-SMI	52.5/41.2	69 (45/24)
Yuri et al. (41)	Japan	2017	Asian	Retrospective	HCC	RFA	HBV 12, HCV 134, others 36	182 (111/71)	70	4.28 years	L3-PMI	6.31/3.91	90 (63/27)
Antonelli et al. (42)	Italy	2018	Caucasian	Retrospective	HCC	Sorafenib	HBV 13, HCV 46, alcohol 16, NASH 11, others 10	96 (75/21)	69	NR	L3-SMI	53 (BMI ≥ 25) or 43 (BMI < 25)/41	47 (28/19)
Ha et al. (43)	Korea	2018	Asian	Retrospective	HCC	NR	HBV 110, HCV 15, Alcohol 27, Unknown 26	178 (141/37)	62.5 (sarcopenia); 58.3 (non-sarcopenia)^b^	NR	L3-SMI	45.8/43	62 (43/19)
Kobayashi et al. (44)	Japan	2018	Asian	Retrospective	HCC	TACE and/or TAI	HBV 11, HCV 50, NBNC 41, alcohol 26, NASH 7, PBC 2, cryptogenic 6	102 (70/32)	69	NR	L3-SMI	42/38	31 (14/17)
Saeki et al. (45)	Japan	2018	Asian	Retrospective	HCC	Sorafenib	HBV 20, HCV 54, alcohol 12, others 14	100 (72/28)	70.6^b^	NR	L3-SMI	42/38	46 (NR)
Shiba et al. (46)	Japan	2018	Asian	Retrospective	HCC	Carbon ion radiotherapy	NR	68 (41/27)	77 (sarcopenia); 74 (non-sarcopenia)	33.5 months	L3-SMI	43.75/41.10	22 (11/11)
Shirai et al. (47)	Japan	2018	Asian	Retrospective	HCC	Hepatectomy	HBV and/or HCV 264, others 138	402 (325/77)	67	NR	L3-PMI	6.36/3.92	134 (NR)
Fujita et al. (48)	Japan	2019	Asian	Retrospective	HCC	TACE	HBV 24, HCV 85, alcohol 39, NASH 26, Others 5	179 (130/49)	72	20.3 months	L3-PMI	6.0/3.4	80 (70/10)
Hamaguchi et al. (49)	Japan	2019	Asian	Retrospective	HCC	Hepatectomy	HBV and/or HCV 392, others 214	606 (484/122)	68	NR	L3-SMI	40.31/30.88	84 (NR)
Imai et al. (50)	Japan	2019	Asian	Retrospective	HCC	Sorafenib	HBV 14, HCV 28, others 19	61 (54/7)	67.3^b^	NR	L3-SMI	42/38	25 (22/3)
Kobayashi et al. (14)	Japan	2019	Asian	Retrospective	HCC	Hepatectomy	HBV and/or HCV 302, others 163	465 (367/98)	67.6^b^	NR	L3-SMI	40.31/30.88	31 (24/7)
Kroh et al. (51)	Germany	2019	Caucasian	Retrospective	HCC	Hepatectomy	NR	70 (49/21)	67.74^b^	NR	L3-SMI	53 (BMI ≥ 25) or 43 (BMI < 25)/41	33 (21/12)
Labeur et al. (52)	Netherlands	2019	Caucasian	Retrospective	HCC	Sorafenib	HBV 46, HCV 44, alcohol 92, NAFLD-NASH 19, other 17, unknown 71	278 (220/58)	64	54.9 months	L3-SMI	53 (BMI ≥ 25) or 43 (BMI < 25)/41	145 (109/36)
Lee et al. (53)	USA	2019	Caucasian	Retrospective	HCC	RT	HBV 113, HCV 14, NBNC 29	156 (128/28)	59	9.3 months	L3-SMI	55/39	99 (81/18)
Yugawa et al. (54)	Japan	2019	Asian	Retrospective	ICC	Hepatectomy	HBV 6, HCV 7	61 (42/19)	69 (sarcopenia); 60 (non-sarcopenia)	NR	L3-PMA	34.6/18.1 cm^2^	30 (20/10)
Akce et al. (17)	USA	2020	Mixed	Retrospective	HCC	Anti-PD-1 antibody	NR	57 (44/13)	66	NR	L3-SMI	43/39	28 (NR)
Bekki et al. (19)	Japan	2020	Asian	Retrospective	HCC	Hepatectomy	HBV 27, HCV 70	139 (110/29)	NR	2.7 years	L3-SMI	52.4/38.5	86 (80/6)
Choi et al. (55)	Korea	2020	Asian	Prospective	HCC	Different treatments	HBV 177, HCV 22, non-viral 39	238 (193/45)	59	31.8 months	L3-PMI	4.98/1.17	135 (130/5)
Ebadi et al. (56)	Canada	2020	Caucasian	Retrospective	HCC	SIRT	HBV 21, HCV 31, alcohol 14, alcohol and HCV 12, NASH 6, others 17	101 (89/12)	62^b^	14 months	L3-SMI	50/39	57 (NR)
Endo et al. (57)	Japan	2020	Asian	Retrospective	HCC	Lenvatinib	HBV 10, HCV 23, alcohol 17, others 13	63 (53/10)	71	8.3 months	L3-SMI	42/38	22 (16/6)
Faron et al. (58)	Germany	2020	Caucasian	Retrospective	HCC	Yttrium-90 radio embolization	HBV 11, HCV 11, alcohol 9, others 27	58 (45/13)	68^b^	250 days	FFMA (MRI)	3582 mm^2^/2301 mm^2^	29 (22/7)
Kotoh et al. (59)	Japan	2020	Asian	Retrospective	HCC	Lenvatinib	HBV 7, HCV 20, Others 26	53 (41/12)	72	NR	Handgrip and L3-SMI	26/18 Kg and 42/38	15 (NR)
Lanza et al. (60)	Italy	2020	Caucasian	Retrospective	HCC	TAE	HBV 7, HCV 65, alcohol 33, NASH 21	142 (110/32)	75	27 months^b^	L3-SMI	55/39	121 (97/24)
Santhakumar et al. (61)	New Zealand	2020	Caucasian	Retrospective	HCC	Hepatectomy	HBV 86, HCV 25, alcohol 6, NAFLD 6, others 24	147 (118/29)	59.1^b^	5.9 years	L3-SMI	46.69/31.03	40 (36/4)
Uojima et al. (62)	Japan	2020	Asian	Retrospective	HCC	Lenvatinib	HBV 19, HCV 34, alcohol 24, NASH 16, others 7	100 (75/25)	71.5^b^	NR	L3-SMI	42/38	59 (42/17)
Wu et al. (63)	China	2020	Asian	Retrospective	HCC	Sorafenib	HBV 84, HCV 23	137 (120/17)	NR	NR	TSM	39.1 for men	18 (male)
Yeh et al. (20)	China	2020	Asian	Retrospective	HCC	RFA	HBV 43, HCV 61, Alcohol 31	136 (78/58)	63.4^b^	3.84 years	L3-PMI	4.24/2.5	22 (16/6)
Deng et al. (64)	China	2021	Asian	Prospective	ICC	Hepatectomy	HBV 46	121 (52/69)	65	16.1 months	L3-PMI	8.6/6.04	53 (NR)
Guichet et al. (65)	USA	2021	Caucasian	Retrospective	HCC	90Y radio embolization.	HBV 26, HCV 43, alcohol 11, NASH 7, cryptogenic 2, PBC 1, unknown 1	82 (65/17)	65	19.6 months^b^	FFMA(MRI)	31.97/28.95 cm^2^	25 (17/8)
Jang et al. (66)	Korea	2021	Asian	Retrospective	HCC	Hepatectomy	HBV 125, HCV 12, non-viral 23	160 (120/40)	55.19^b^	7.9 years	L3-PMI	3.33/2.38	28 (17/11)
Li et al. (67)	China	2021	Asian	Retrospective	ICC	Hepatectomy	HBV 136, HCV 2, hepatolithiasis 83	460 (223/237)	58	NR	L3-SMI	42.6/37.8	281 (137/144)
Liao et al. (68)	China	2021	Asian	Retrospective	HCC	Hepatectomy	HBV 385	452 (386/66)	53.15^b^	NR	L3-SMI	40.86/30.71	NR
Saeki et al. (69)	Japan	2021	Asian	Retrospective	HCC	Sorafenib	HBV 80, HCV 175, HBV + HCV 2, NBNC 99	356 (287/69)	69.5	NR	L3-SMI	45/38	175 (NR)
Salman et al. (70)	Egypt	2021	Caucasian	Prospective	HCC	RFA	HCV 97	97 (72/25)	53.4^b^	NR	L3-SMI	53 (BMI ≥ 25) or 43 (BMI < 25)/41	42 (28/14)
Yoshio et al. (71)	Japan	2021	Asian	Retrospective	HCC	Hepatectomy	HBV 61, HCV 86, NBNC 87	234 (183/51)	67.4	NR	L3-SMI	42/38	82 (NR)
Chien et al. (72)	China	2022	Asian	Retrospective	HCC	TACE	HBV 141, HCV 110	260 (192/68)	64^b^	NR	L3-PMI	6.36/3.92	130 (103/27)
Dong et al. (73)	China	2022	Asian	Retrospective	HCC	Lenvatinib	HBV 35, HCV 3, NBNC 2	40 (37/3)	59	9.2 months	L3-SMI	42/38	23 (20/3)
Fujita et al. (74)	Japan	2022	Asian	Retrospective	HCC	Lenvatinib	HBV 28, HCV 35, Alcohol 37, NAFLD 26, Others 4	130 (107/23)	70	NR	L3-PMI	6.0/3.4	63 (58/5)
Hayashi et al. (75)	Japan	2022	Asian	Retrospective	HCC	Hepatectomy	HBV 49, HCV 120	303 (221/82)	72 (sarcopenia); 70 (non-sarcopenia)	NR	L3-SMI	52.4/38.9	106 (96/10)
Hou et al. (76)	China	2022	Asian	Retrospective	Combined hepatocellular carcinoma and cholangio carcinoma (cHCC-CC)	Hepatectomy	HBV 119, HCV 3	153 (128/25)	NR	41.3 months	L3-PMI	5.42/4.05	77 (64/13)
Kim et al. (77)	South Korea	2022	Asian	Retrospective	HCC	Hepatectomy	HBV or HCV 120, others 39	159 (133/26)	59.3^b^	45 months	L3-SMI	52.4/38.5	74 (68/6)
Roth et al. (78)	France	2022	Caucasian	Retrospective	HCC	TACE	HBV 21, HCV 64, alcohol 138, NASH 66	225 (200/25)	65	NR	L3-SMI	50/39	130 (120/10)
Tamai et al. (79)	Japan	2022	Asian	Retrospective	HCC	Hepatectomy or RFA	HBV 24, HCV 92, NASH 29, alcohol 29, others 7	181 (129/52)	71.4^b^	39.2 ± 13.7 months	L3-PMI	6.36/3.92	100 (73/27)
Xiao et al. (80)	China	2022	Asian	Retrospective	Primary liver cancer	ICIs	HBV 153, HCV 3, HBV + HCV 2, none 14	172 (149/23)	51.4^b^	9 months	L3-SMI	53 (BMI ≥ 25) or 43 (BMI < 25)/41	68 (52/16)
Yang et al. (81)	China	2022	Asian	Retrospective	HCC	SBRT	HBV 71, HCV 36, HBV + HCV 8, non-virus 22	137 (106/31)	63.8^b^	14.1 months	L3-SMI	49/31	67 (63/4)
Zhang et al. (82)	China	2022	Asian	Retrospective	HCC	TACE	HBV 194, others 34	228 (175/53)	58.9^b^	22.3 months	L3-SMI	45.95/33.96	89 (76/13)

NR, not reported; L3-SMI, skeletal muscle index at the level of third vertebra; L3-PMI, psoas muscle index at the level of third vertebra; L3-PMA, psoas muscle mass area at the level of third vertebra; L3-TPA, total psoas area at the level of third vertebra; FFMA, fat-free muscle area; TSM, total skeletal muscle; SO, sarcopenic obesity; LDLT, living donor liver transplantation; OLT, orthotopic liver transplantation; HCC, hepatocellular carcinoma; ICC, intrahepatic cholangiocarcinoma; RFA, radiofrequency ablation; TAI, transcatheter arterial infusion; TACE, transcatheter arterial chemoembolization; RT, radiotherapy; SIRT, selective internal radiation therapy; TAE, transarterial embolization; SBRT, stereotactic body radiotherapy; ICIs, immune checkpoint inhibitors; HBV, hepatitis B virus; HCV, hepatitis C virus; NASH, non-alcoholic steatohepatitis; NAFLD, non-alcoholic fatty liver disease; NBNC, without HBV or HCV; PBC, primary biliary cholangitis.

a Values are median unless indicated otherwise.

b Values are mean.

Of the 11,970 patients, 8,919 were men (74.51%) and 3,051 were women (25.49%), with a median (or mean) age of 51.4–77 years. The median or mean age was not reported in three studies (19, 63, 76). Fifty-six of the 64 studies involved patients with HCC. Seventeen studies enrolled patients with resectable HCC; the remaining treatment regimens included transarterial chemoembolization (TACE), liver transplantation (LT), radiofrequency ablation (RFA), and administration of kinase inhibitors. The curative treatment included liver resection, RFA, and LT, whereas the palliative treatment included intra-arterial chemoembolization, administration of kinase inhibitors, and systemic chemotherapy. Viral hepatitis was the most common etiology of liver cancer, with HCV as the primary cause of viral hepatitis in most included studies (*n* = 36). The other causes of liver cancer include alcohol consumption, non-alcoholic steatohepatitis (NASH), and others. Six studies did not report the etiology of liver cancer. Thirty of the 64 studies reported the duration of follow-up (median or mean), which ranged from 8.3 months to 7.9 years.

### 3.2. Quality assessment

The quality of the included studies was determined by NOS. Patient selection, comparability, and outcomes were used to evaluate the methodological quality of each study. Supplementary Table S2 shows the evaluation of the quality of the included studies. Based on the NOS score of ≥7, all the included studies were considered to have high quality.

### 3.3. Definition of sarcopenia and SO

The approaches to identifying sarcopenia and SO in patients were different. In most studies (*n* = 61), the areas of visceral/subcutaneous fat and skeletal muscle were determined by a transverse analysis at the level of the third lumbar vertebra (L3). In 45 studies, sarcopenia was defined by the sex-specific cutoff values of the L3-SMI (skeletal muscle index, cm^2^/m^2^), which showed a slight variation in those studies (shown in Table 1). Seven studies used different values to define sarcopenia depending on body mass index (BMI) (24, 37, 42, 51, 52, 70, 80). The majority of studies (44 studies) used cutoff values between 40 and 55 cm^2^/m^2^ in men. Nine studies evaluated sarcopenia based on the L3-PMI (psoas muscle index, cm^2^/m^2^), and one study used the total skeletal muscle mass (TSM) index. The other two studies defined sarcopenia as low fat-free muscle area (FFMA) based on MRI evaluation. According to these sex-specific cutoff values, 3,957 patients were diagnosed with sarcopenia in 62 studies, yielding a pooled prevalence of 43.2% (95% CI: 37.8%–48.5%). Among the 50 studies reporting the prevalence according to gender, a slight difference in prevalence was observed between female and male patients, with a higher pooled prevalence of 45% among men compared to 42.2% among women.

Four of the 64 studies reported the impact of SO on the survival of liver cancer patients. Regarding the definition of SO, Itoh et al. (33) used low skeletal muscle mass-to-visceral fat area ratio (SVR) to define SO, while Kobayashi et al. (14), Kroh et al. (51), and Liao et al. (68) used the co-existence of sarcopenia and obesity to define SO. However, variations were noted in the definition of obesity. Obesity was defined as the area of visceral adipose tissue at the level of the third lumbar vertebra ≥100 cm^2^ in both men and women by Kobayashi et al., while the patients were considered obese if their BMI was ≥25 kg/m^2^ in Liao's study; in Kroh's study, obesity was defined by categorizing individuals within the highest two quintiles body fat percentage for men and women.

### 3.4. OS in sarcopenic patients

**Figure 2:**
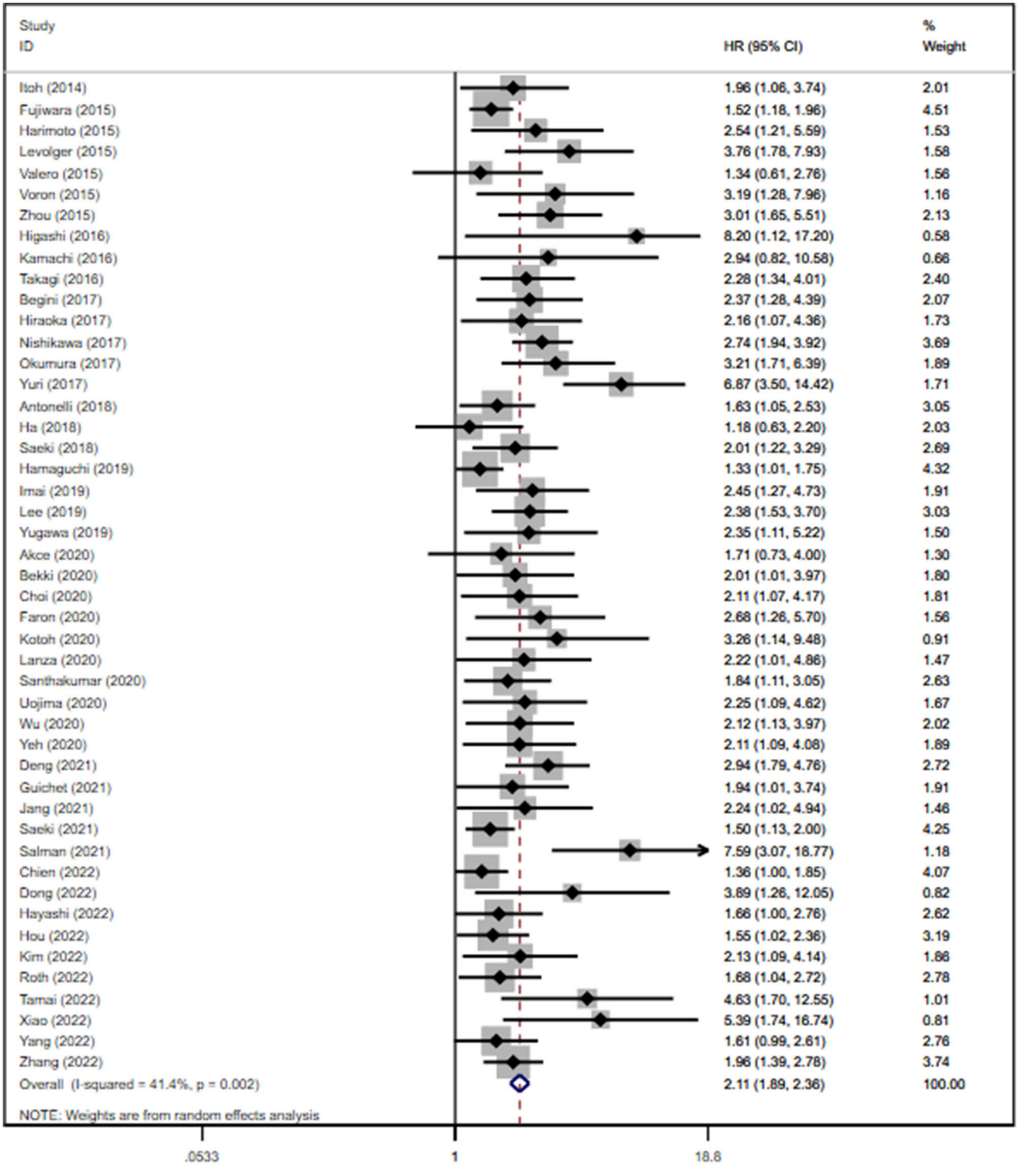
Forest plot of the pooled adjusted hazard ratios for the association between sarcopenia and overall survival in patients with primary liver cancer.

Not all the eligible studies reported the HRs of OS. After calculating the data in a univariate analysis of 51 (*n* = 9,615) studies, we found that sarcopenia was related to lower OS, with a pooled unadjusted HR of 1.94 (95% CI: 1.76–2.13, *P* < 0.0001; shown in Supplementary Figure S1). A multivariate analysis of 47 studies (*n* = 8,285) revealed that the risk of death in sarcopenic patients was 2.11-fold higher than that in non-sarcopenic patients (95% CI: 1.89–2.36, *P* < 0.0001; shown in Figure 2). In most studies, HR was adjusted for age, gender, BMI, alpha-fetoprotein (AFP), tumor stage, and comorbidity. According to sensitivity analysis, no individual study had a significant impact on the pooled unadjusted HR and adjusted HR, thus indicating that the results were robust (Supplementary Figure S2). The test for heterogeneity showed a moderate result for both univariate and multivariate analyses (*I*^2^ = 44.5% and *P* < 0.1 for the unadjusted HRs; *I*^2^ = 41.4% and *P* < 0.1 for the adjusted HRs). The funnel plots were asymmetric in both univariate and multivariate analyses (Supplementary Figures S3, S4). Potential publication bias was significant for both unadjusted HRs (*P* = 0.000 < 0.05) and adjusted HRs (*P* = 0.000 < 0.05), according to Egger's test. Therefore, we used the trim-and-fill method by imputing the potential unpublished articles for unadjusted HRs and adjusted HRs to achieve symmetry in the funnel plot (Supplementary Figures S3, S4). The pooled unadjusted and adjusted HRs were 5.42 (95% CI: 4.60–6.50) and 5.251 (95% CI: 4.53–6.87), respectively. We further conducted a subgroup analysis on OS for the adjusted HRs as designed previously. Interestingly, the results showed that sarcopenia was consistently correlated with poor OS across all the analyzed subgroups. Sarcopenia (vs. non-sarcopenia) was associated with low OS in both Asian and non-Asian regions with summary adjusted HR of 2.10 (95% CI: 1.84–2.39) and 2.18 (95% CI: 1.78–2.66), respectively; in patients with HCC and ICC (summary adjusted HR: 2.07, 95% CI: 1.84–2.33; summary adjusted HR: 2.91, 95% CI: 2.15–3.94, respectively); in patients treated with curative and palliative therapies (pooled adjusted HR: 2.45, 95% CI: 2.01–3.00; pooled adjusted HR: 1.93, 95% CI: 171–2.18, respectively); in patients defined by L3-SMI, L3-PMI, and FFMA (MRI; pooled adjusted HR: 2.07, 95% CI: 1.82–2.35; pooled adjusted HR: 2.36, 95% CI: 1.68–3.31; pooled adjusted HR: 2.23, 95% CI: 1.36–3.65, respectively); and in patients with only HCV-related and other causes (pooled adjusted HR: 5.28, 95% CI: 2.14–13.04; pooled adjusted HR: 2.06, 95% CI: 1.85–2.29, respectively; shown in Supplementary Table S3).

### 3.5. OS in patients with SO

**Figure 3:**
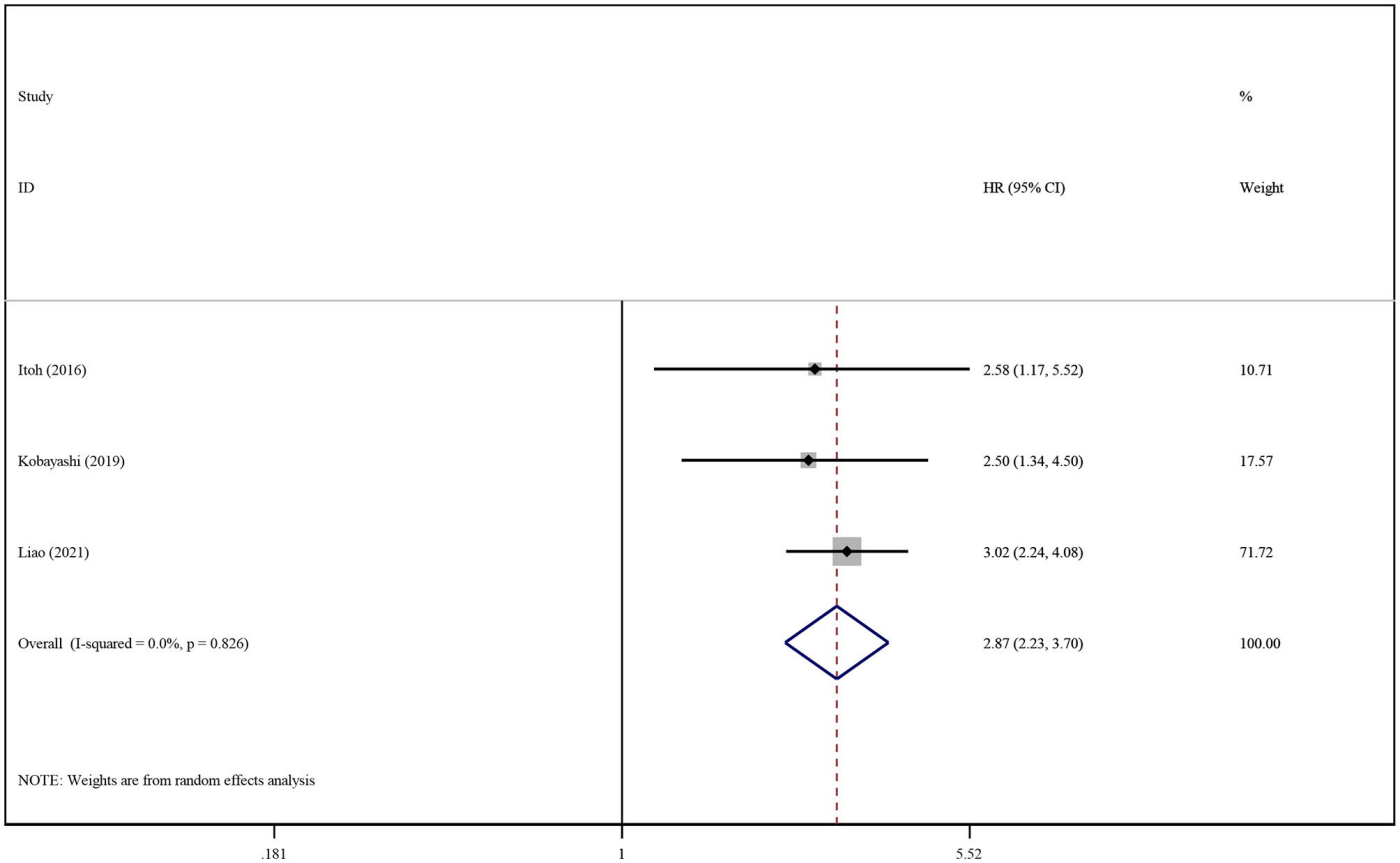
Forest plot of the pooled adjusted hazard ratios for the association between sarcopenic obesity and overall survival in patients with primary liver cancer.

Only four studies reported statistical data regarding the influence of SO on patient survival (14, 33, 51, 68). The prevalence of SO varied greatly among the studies, ranging from 6.67% to 30.00% (characteristics shown in Table 1). We conducted respective analyses of studies reporting unadjusted and adjusted HRs for OS. Patients with SO had a higher risk of death than those without SO (pooled unadjusted HR: 2.08. 95% CI 1.67–2.60, *P* < 0.0001; pooled adjusted HR: 2.87; 95% CI: 2.23–3.70, *P* < 0.0001), as shown in Supplementary Figure S5 and Figure 3. The subgroup analysis was not conducted because of the limited number of studies reporting SO data.

### 3.6. RFS/DFS of the study patients

**Figure 4:**
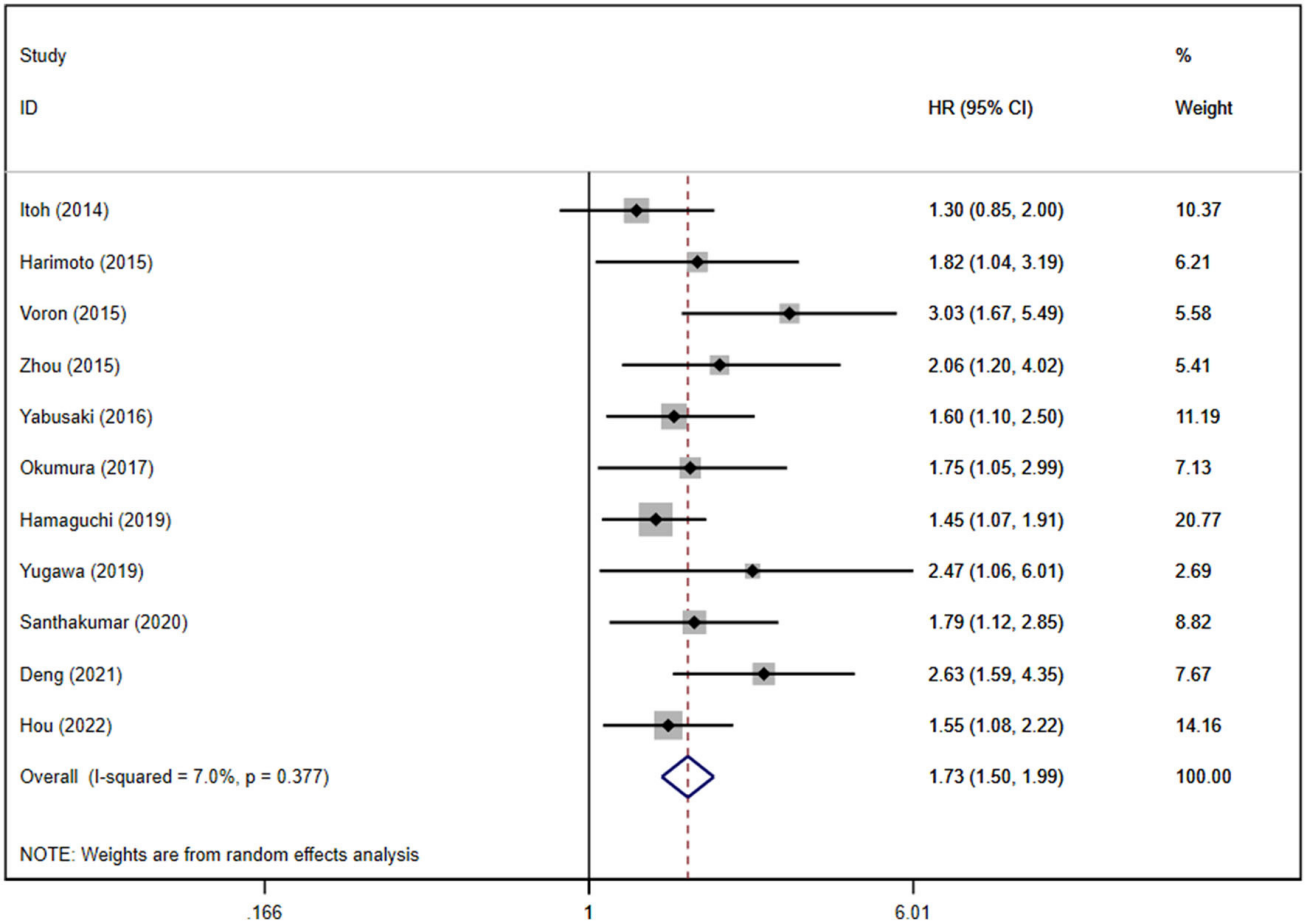
Forest plot of the pooled adjusted hazard ratios for the association between sarcopenia and recurrence-free survival or disease-free survival in patients with primary liver cancer.

We conducted separate analyses of studies reporting unadjusted and adjusted HRs for RFS/DFS. A univariate analysis of 16 studies showed poor RFS/DFS in patients with sarcopenia (HR: 1.74, 95% CI: 1.50–2.02, *P* < 0.0001; Supplementary Figure S6), while a multivariate analysis of 11 studies showed a similar result (HR: 1.73, 95% CI: 1.50–1.99, *P* < 0.0001; Figure 4).

Moderate heterogeneity was observed in univariate analysis, while mild heterogeneity was noted in multivariate analysis (*P* = 0.027 and *I*^2^ = 44.8% for the unadjusted HRs; *P* = 0.377 and *I*^2^ = 7.0% for the adjusted HRs). Publication bias was not found in the univariate analysis (Egger's test, *P* = 0.587), whereas the multivariate analysis showed the existence of publication bias (Supplementary Figure S7). We further conducted a subgroup analysis on RFS/DFS for the adjusted HRs according to ethnicity, type of liver cancer, and sarcopenia definitions. However, we did not perform a subgroup analysis on RFS/DFS for the adjusted HRs based on treatment modalities because all subjects were treated by hepatectomy. The results are shown in Supplementary Table S4.

**Figure 5:**
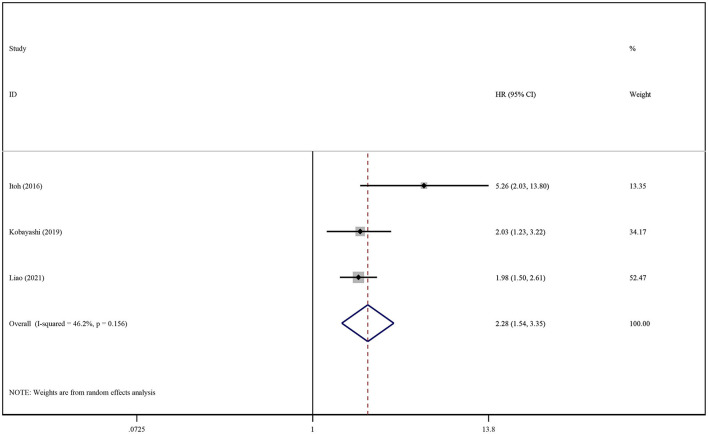
Forest plot of the pooled adjusted hazard ratios for the association between sarcopenic obesity and recurrence-free survival or disease-free survival in patients with primary liver cancer.

The association between SO existence and its influence on RFS/DFS in HCC was analyzed in the same manner. The summary of crude and adjusted HRs were 1.80 (95% CI: 1.33–2.44, *P* < 0.001; Supplementary Figure S8) and 2.28 (95% CI: 1.54–3.35, *P* < 0.001; Figure 5), respectively; this indicated that SO was associated with a lower RFS/DFS.

## 4. Discussion

In the present meta-analysis, we analyzed 64 studies comprising 11,970 participants diagnosed with primary liver cancer. Sarcopenia is a common disorder in this population, with a pooled prevalence of 43.2%, and it is more prevalent in men. The results revealed a robust association between sarcopenia and/or SO and patient survival. A strong relationship between sarcopenia and adverse clinical outcomes in cancer patients has been reported in prior studies, including depression (83), risk of fall (84), higher risk of complications (85), and cancer recurrence and mortality (9, 13, 15, 86). In 2022, a meta-analysis comprising 280 publications involving 81,814 patients with solid tumors demonstrated that sarcopenia is a prevalent condition in oncological patients with a prevalence of 35.3%; moreover, it is particularly higher in patients with specific cancer types such as esophageal cancer, urothelial cancer, cholangiocarcinoma, prostate cancer, and thyroid cancer (87). A recent study reported that SO affected 20% of cancer patients, demonstrating a significant association with various poor outcomes in cancer patients, such as low OS, RFS, and longer length of hospital stay, particularly in patients with oropharyngeal cancer and liver cancer (88). A previous systematic review of three articles involving 1,515 liver transplant recipients demonstrated a two-fold increase in mortality rates linked to pre-transplant SO (89).

The underlying mechanisms of sarcopenia and SO are poorly understood. According to prior studies, sarcopenia and SO are multifactorial conditions. The key mechanisms of sarcopenia include aging, inflammation, hormonal changes, inactivity, and low-protein intake. The available evidence indicates that the elderly population, especially individuals aged 65 years and above, is susceptible to anabolic resistance due to decreased availability of post-prandial amino acid, diminished muscle perfusion, and decreased digestive ability caused by the sequestration of amino acids in the splanchnic region (90). Body fat increases with age until 70 years. This accumulation of body fat activates macrophages, mast cells, and T lymphocytes, resulting in the secretion of inflammatory factors such as tumor necrosis factor (TNF), leptin, IL-6, and growth hormone (GH), which induces an array of inflammatory responses (91). Inflammatory factors such as TNF-α and IL-6 facilitate skeletal muscle wasting; the former directly catabolizes skeletal muscle, leading to increased gluconeogenesis, proteolysis, and upregulation of uncoupling proteins (UCPs) 2 and 3 in cachectic skeletal muscle, while the latter suppresses protein synthesis in muscle cells by the Janus kinase signaling pathway (92). Testosterone not only modulates inflammation in skeletal muscle by activating satellite cells to promote muscle regeneration but also increases the utilization of amino acids and androgen receptor expression in skeletal muscle to promote muscle protein synthesis; however, the levels of testosterone decline with age, which is likely to have a negative effect on muscle mass (93). Inactivity can affect muscle metabolism, further exacerbating the catabolic response and decreasing muscle protein synthesis (11). Prior studies have shown that exercise can improve muscle strength and mass, and both resistance training and aerobic exercise are beneficial to sarcopenia (94, 95). A previous review elaborated on the mechanisms of SO, which included lipotoxicity, adipose tissue inflammation, adipose tissue dysfunction, insulin resistance, and systemic chronic sterile low-grade inflammation. The authors proposed intricate interactions between adipose tissue and skeletal muscle, leading to the establishment of a detrimental vicious circle as individual's age, resulting in chronic low-grade local inflammation and systemic inflammation (91). Currently, there is a lack of specific medicines to treat SO. Lifestyle intervention is the most important method to treat SO, including calorie restriction; aerobic exercise; resistance exercise; and supplementation of protein, calcium, and vitamin D (93). Exercise intervention can positively change body composition and improve body weight, BMI, fat mass, body fat percentage, grip strength, and walking speed in the SO population; nutritional intervention can decrease fat mass with no improvement in grip strength (96).

To the best of our knowledge, this is the first meta-analysis to demonstrate a significant relationship between sarcopenia, SO, and survival in patients with primary liver cancer in a large sample. The study included patients who underwent either curative or palliative treatment. A strength of this meta-analysis is that the study population included patients with HCC and ICC for the first time. Although a prior meta-analysis of studies published before 2017 examined the association between sarcopenia and mortality in HCC patients, one of the studies included not only patients with HCC but also metastatic liver cancer (97). Another meta-analysis included cohorts that overlapped (98), while one study focused only on the prognosis of sarcopenia in HCC patients treated with sorafenib or lenvatinib (99).

Additionally, in the present meta-analysis, a comprehensive search was performed, and studies with overlapping cohorts were excluded. Consequently, our meta-analysis added 40 additional studies that were not analyzed in previous meta-analyses, thus contributing to 64 included studies. We found a correlation between SO and decreased OS. There is, however, a lack of extensive research on the effect of SO on survival in patients diagnosed with liver cancer.

Our study has several limitations. First, this study was constrained by insufficient data from each of the included studies, which is inherent to the nature of meta-analysis. Additionally, not all studies reported an adjusted HR, which could potentially restrict our ability to determine the precise magnitude of the mortality risk between sarcopenic and non-sarcopenic patients. Second, the selection of adjusted variables for the multivariate Cox regression models varied among the studies. Third, the significant results of Egger's tests indicated the presence of publication bias.

Sarcopenia was defined as the presence of both low muscle mass and impaired muscle function, according to EWGSOP2 (2). However, the diagnosis of sarcopenia remains controversial. On the one hand, there is a lack of standardized and feasible methods to measure muscle function or physical performance. Importantly, this limitation applies not only to the current meta-analysis but also to all existing studies that have investigated the impact of sarcopenia and/or SO on patients with malignant carcinomas. On the other hand, only prospective cohort studies are likely to document muscle function. Therefore, all the included studies can only partially define sarcopenia by measuring muscle mass, depending on CT/MRI images at the L3 level, which can be easily obtained from medical records. Hence, the retrospective nature of the included studies is recognized as a limitation of the current study. Given that the majority of the included studies were retrospective cohort studies, it is probable that the results were influenced by selection bias, as only patients who underwent CT scans were included. Furthermore, several methods were used to measure muscle mass, and the studies evaluating SMI and PMI at the L3 level based on CT imaging were included in the present meta-analysis; this may partially result in heterogeneity. We observed slight variations in the actual sex-specific cutoff values used in different studies. In some studies, the authors predefined the thresholds, whereas other studies derived these thresholds from their own study population to calculate the cutoff values. The thresholds were higher in European and American populations than in Asian populations, which might be explained by the fact that previous western studies were deemed unsuitable for Asian patients. Additionally, different cutoff values might change the magnitude of the association between sarcopenia and survival. Hence, we performed a subgroup analysis to overcome these limitations. The subgroup analysis showed a significant association between sarcopenia and poor OS and RFS/DFS across all the analyzed subgroups. Further prospective studies evaluating both muscle mass and muscle function are necessary to accurately and timely identify sarcopenic patients and to better clarify the relationship between muscle loss and survival.

Unlike previous studies examining the association between sarcopenia and survival in liver cancer patients, there is limited research on the association between SO and survival in liver cancer patients. Of the five studies searched, one study was excluded because it involved patients with other diseases, such as liver cirrhosis and cholestatic diseases (100). Furthermore, the four studies defined SO by using different approaches. Hence, further studies are required to confirm the association between SO and survival.

## 5. Conclusion

Sarcopenia and SO exhibited a significant association with reduced OS and RFS/DFS in patients with liver cancer. Additionally, the evaluation of sarcopenia and SO needs a consensus regarding their definitions and the utilization of appropriate cutoff values. We suggest that patients with liver cancer should undergo initial evaluation for sarcopenia and SO and receive regular monitoring because of poor prognosis. Further prospective studies are required to integrate sarcopenia and SO into an established prognostic scale specifically tailored for patients with liver cancer.

## Data availability statement

The original contributions presented in the study are included in the article/Supplementary material, further inquiries can be directed to the corresponding author.

## Author contributions

XL and ST: study design. XL and XH: methodology and acquisition of data. XL: formal analysis and writing—original draft preparation. XL, XH, LL, and ST: writing—review and editing. All authors have read and agreed to the published version of the manuscript.
