# Supplementary material for: Impact of sarcopenia and sarcopenic obesity on survival in patients with primary liver cancer: a systematic review and meta-analysis
Source: Front Nutr. 2023 Oct 19;10:1233973. doi: 10.3389/fnut.2023.1233973 (PMC10620805; doi:10.3389/fnut.2023.1233973)
Supplement: Supplementary file 1 [file Data_Sheet_1.docx]

**Supplementary Figures:**


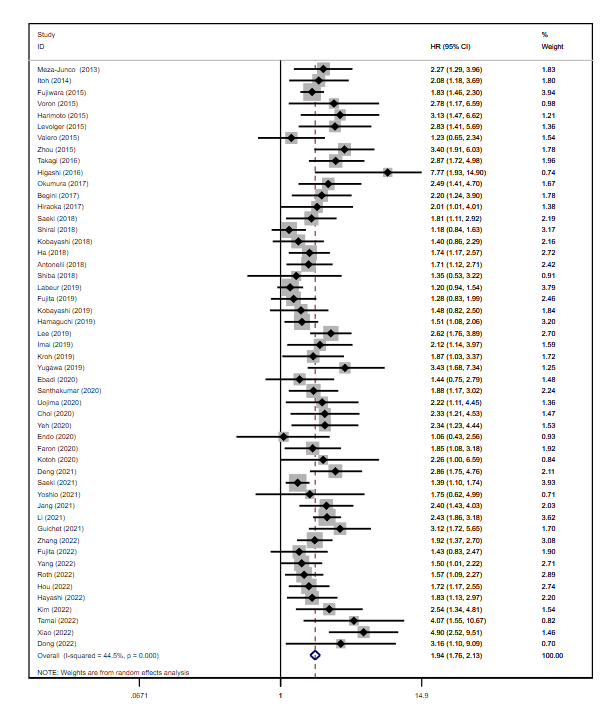


**Figure S1** Forest plot of the pooled unadjusted hazard ratios for association between sarcopenia and overall survival in patients with primary liver cancer.


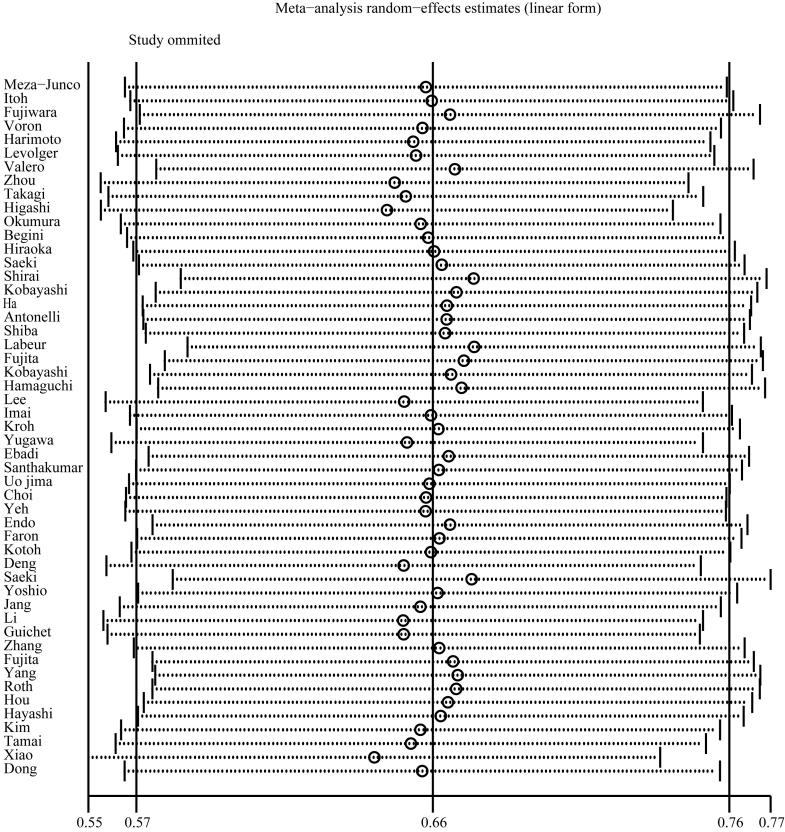

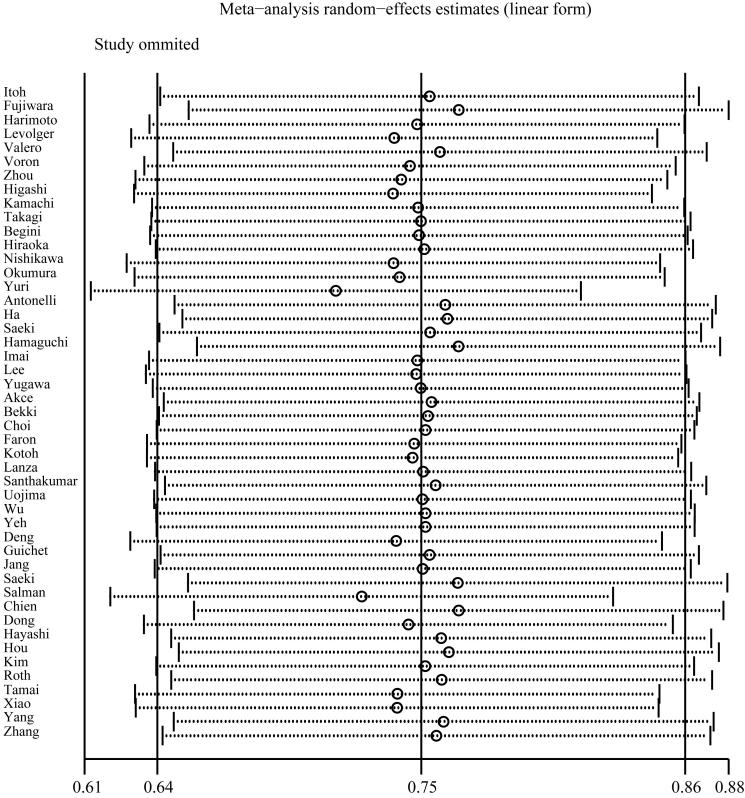


a b

**Figure S2** a: Sensitivity analysis for crude HR of overall survival; b: Sensitivity analysis for adjusted HR of overall survival.

a b

**Figure S3** Funnel plot for assessing potential publication bias. a: Funnel plot for crude HR of OS plotted prior to application of the trim-and-fill method; b: Funnel plot for crude HR of OS plotted after application of the trim-and-fill method.

a b

**Figure S4** Funnel plot for assessing potential publication bias. a: Funnel plot for adjusted HR of OS plotted prior to application of the trim-and-fill method; b: Funnel plot for adjusted HR of OS plotted after application of the trim-and-fill method.


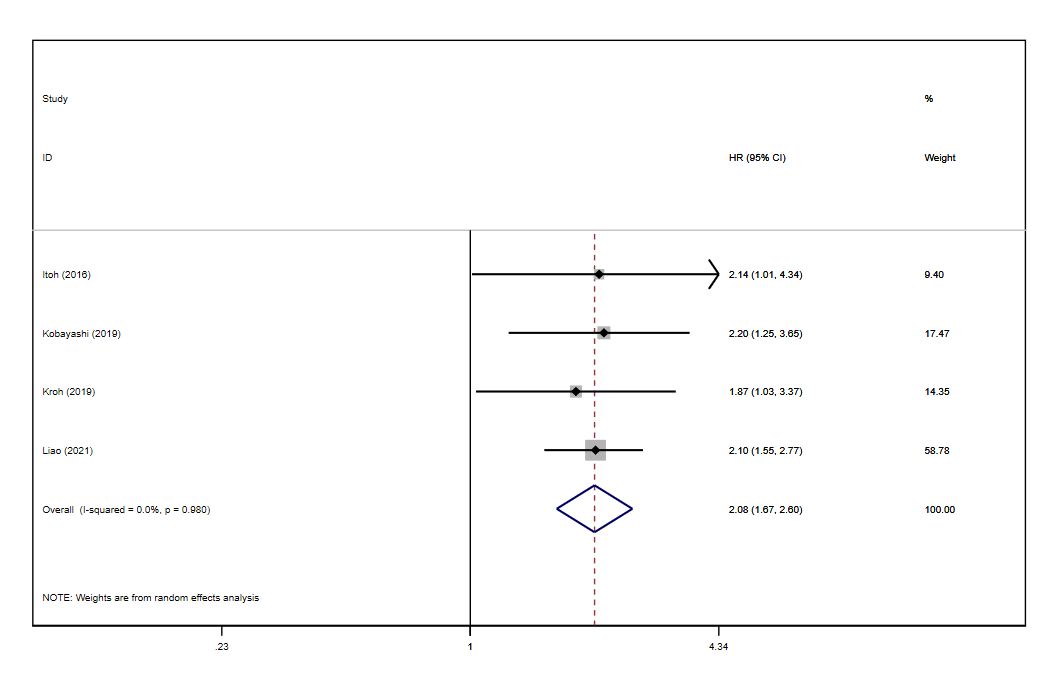


**Figure S5** Forest plot of the pooled unadjusted hazard ratios for association between sarcopenic obesity and overall survival in patients with primary liver cancer.


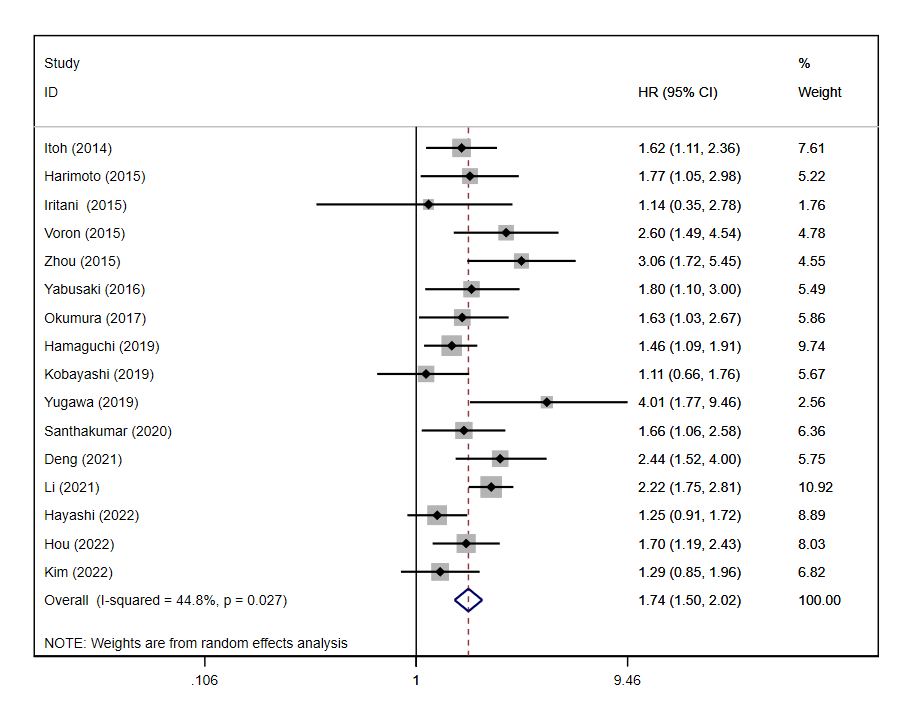


**Figure S6** Forest plot of the pooled unadjusted hazard ratios for association between sarcopenia and recurrence-free or disease-free survival in patients with primary liver cancer.


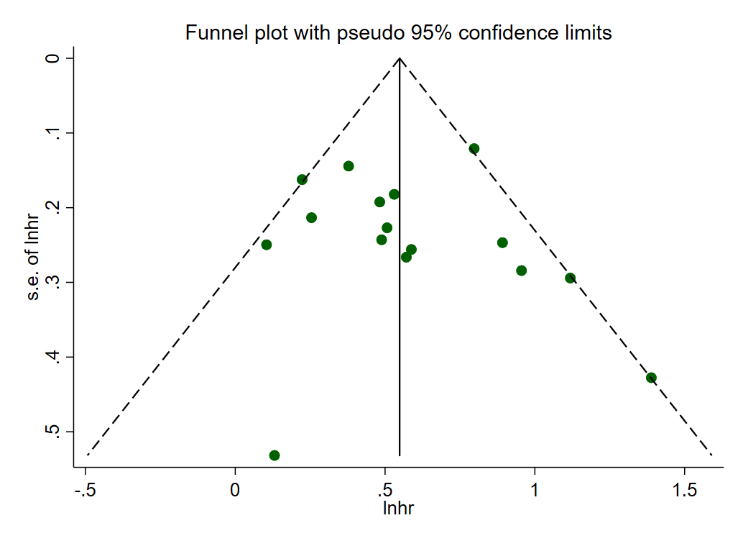

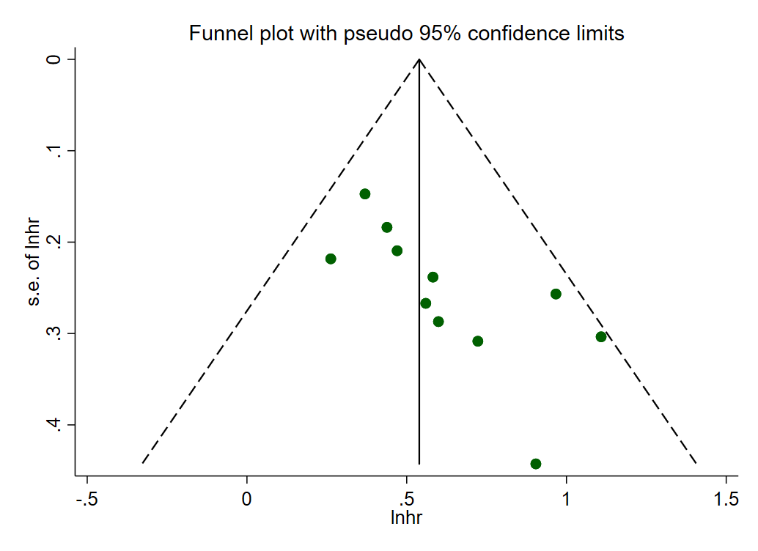


a b

**Figure S7** Funnel plot for assessing potential publication bias. a: Funnel plot for crude HR of RFS/DFS; b: Funnel plot for adjusted HR of RFS/DFS.

**Figure S8** Forest plot of the pooled unadjusted hazard ratios for association between sarcopenic obesity and recurrence-free or disease-free survival in patients with primary liver cancer.
