# Supplementary material for: Impact of sarcopenia and sarcopenic obesity on survival in patients with primary liver cancer: a systematic review and meta-analysis
Source: Front Nutr. 2023 Oct 19;10:1233973. doi: 10.3389/fnut.2023.1233973 (PMC10620805; doi:10.3389/fnut.2023.1233973)
Supplement: Supplementary file 2 [file Data_Sheet_2.docx]

**Supplementary tables:**

**Table S1.** Detailed search strategy

| **Search strategy** | |  | **Results** |
| --- | --- | --- | --- |
| PubMed |  | |  |
| #1 | "Sarcopenia"[MeSH Terms] | | 8199 |
| #2 | "sarcopeni*"[Title/Abstract] | | 14655 |
| #3 | "myopeni*"[Title/Abstract] | | 67 |
| #4 | "muscle loss"[Title/Abstract] | | 2575 |
| #5 | "muscle depletion"[Title/Abstract] | | 224 |
| #6 | "muscle wasting"[Title/Abstract] | | 5882 |
| #7 | "muscle reduction"[Title/Abstract] | | 66 |
| #8 | "reduced muscle"[Title/Abstract] | | 2512 |
| #9 | "muscular weakness"[Title/Abstract] | | 2044 |
| #10 | "muscle weakness"[Title/Abstract] | | 17204 |
| #11 | "frail*"[Title/Abstract] | | 32756 |
| #12 | "frailty"[Title/Abstract] | | 22488 |
| #13 | "low muscle mass"[Title/Abstract] | | 1205 |
| #14 | "low muscle function"[Title/Abstract] | | 52 |
| #15 | #1 OR #2 OR #3 OR #4 OR #5 OR #6 OR #7 OR #8 OR #9 OR #10 OR #11 OR #12 OR #13 OR #14 | | 73010 |
| #16 | "carcinoma, hepatocellular"[MeSH Terms] | | 100501 |
| #17 | "hepatocellular carcinoma"[Title/Abstract] | | 109512 |
| #18 | "hcc"[Title/Abstract] | | 71756 |
| #19 | "hepatic cell carcinoma"[Title/Abstract] | | 82 |
| #20 | "liver neoplasm"[Title/Abstract] | | 519 |
| #21 | "hepatoma"[Title/Abstract] | | 28700 |
| #22 | "primary liver carcinoma"[Title/Abstract] | | 345 |
| #23 | "liver cell carcinoma"[Title/Abstract] | | 307 |
| #24 | "liver cancer"[Title/Abstract] | | 27164 |
| #25 | #16 OR #17 OR #18 OR #19 OR #20 OR #21 OR #22 OR #23 OR #24 | | 171449 |
| #26 | #15 AND #25 | | 377 |
| #27 | #26 AND (humans[Filter]) AND (english[Filter]) | | 210 |
|  |  | |  |
| Cochrane |  | |  |
| #1 | MeSH descriptor: [Sarcopenia] explode all trees | | 644 |
| #2 | (sarcopeni*):ab,ti OR (myopeni*):ab,ti OR (muscle loss):ab,ti OR (muscle depletion):ab,ti OR (muscle wasting):ab,ti OR (muscle reduction):ab,ti OR (reduced muscle):ab,ti OR (muscular weakness):ab,ti OR (muscle weakness):ab,ti OR (frail*):ab,ti OR (frailty):ab,ti OR (low muscle mass):ab,ti OR (low muscle function):ab,ti | | 25495 |
| #3 | #1 OR #2 | | 25604 |
| #4 | MeSH descriptor: [carcinoma, hepatocellular] explode all trees | | 2040 |
| #5 | (hepatocellular carcinoma):ab,ti OR (hcc):ab,ti OR (hepatic cell carcinoma):ab,ti OR (liver neoplasm):ab,ti OR (hepatoma):ab,ti OR (primary liver carcinoma):ab,ti OR (liver cell carcinoma):ab,ti OR (liver cancer):ab,ti | | 13255 |
| #6 | #4 or #5 | | 13414 |
| #7 | #3 AND #6 | | 133 |
|  |  | |  |
| Embase |  | |  |
| #1 | sarcopenia'/exp | | 17573 |
| #2 | sarcopeni*':ab,ti OR 'myopeni*':ab,ti OR 'muscle loss':ab,ti OR 'muscle depletion':ab,ti OR 'muscle wasting':ab,ti OR 'muscle reduction':ab,ti OR 'reduced muscle':ab,ti OR 'muscular weakness':ab,ti OR 'muscle weakness':ab,ti OR 'frail*':ab,ti OR 'low muscle mass':ab,ti OR 'frailty':ab,ti OR 'low muscle function':ab,ti | | 107418 |
| #3 | #1 OR #2 | | 109811 |
| #4 | carcinoma, hepatocellular'/exp | | 196517 |
| #5 | hepatocellular carcinoma':ab,ti OR 'hcc':ab,ti OR 'hepatic cell carcinoma':ab,ti OR 'liver neoplasm':ab,ti OR 'hepatoma':ab,ti OR 'primary liver carcinoma':ab,ti OR 'liver cell carcinoma':ab,ti OR 'liver cancer':ab,ti | | 216927 |
| #6 | #4 OR #5 | | 263042 |
| #7 | #3 AND #6 | | 913 |
| #8 | #7 AND 'human'/de | | 834 |
|  |  | |  |
| Web of science |  | |  |
| #1 | TS=(sarcopenia OR sarcopeni* OR myopeni* OR muscle loss OR muscle depletion OR muscle wasting OR muscle reduction OR reduced muscle OR muscular weakness OR muscle weakness OR frail* OR frailty OR low muscle mass OR low muscle function) | | 277786 |
| #2 | TS=(carcinoma, hepatocellular OR hepatocellular carcinoma OR hcc OR hepatic cell carcinoma OR liver neoplasm OR hepatoma OR primary liver carcinoma OR liver cell carcinoma OR liver cancer) | | 263188 |
| #3 | #1 AND #2 | | 2365 |
| #4 | TS=(mouse OR rat OR mice OR rabbit) | | 2348696 |
| #5 | #3 NOT #4 | | 1465 |
| #6 | #3 NOT #4 and English (Languages) | | 1445 |

**Table S2.** Quality assessment by using the Newcastle-Ottawa Scale for the included studies.

| **Author** | **Year** | **Selection** | | | | **Comparability** | **Outcome** | | | **Quality (9 point)** |
| --- | --- | --- | --- | --- | --- | --- | --- | --- | --- | --- |
|  |  | **Representative ness of the exposed cohort (1 point)** | **Selection of the non exposed cohort (1 point)** | **Ascertainment of exposure (1 point)** | **Demonstration that outcome of interest was not present at start of study (1 point)** | **Comparability of cohorts on the basis of the design or analysis (2 point)** | **Assessment of outcome (1 point)** | **Was follow-up long enough for outcomes to occur (1 point)** | **Adequacy of follow up of cohorts (1 point)** |  |
|  |  |  |  |  |  |  |  |  |  |  |
|  |  |  |  |  |  |  |  |  |  |  |
|  |  |  |  |  |  |  |  |  |  |  |
|  |  |  |  |  |  |  |  |  |  |  |
| Meza-Junco | 2013 | 1 | 1 | 1 | 1 | 2 | 1 | 0 | 1 | 8 |
| Itoh | 2014 | 1 | 1 | 1 | 1 | 2 | 1 | 1 | 1 | 9 |
| Fujiwara | 2015 | 1 | 1 | 1 | 1 | 2 | 1 | 1 | 1 | 9 |
| Harimoto | 2015 | 1 | 1 | 1 | 1 | 1 | 1 | 1 | 1 | 8 |
| Iritani | 2015 | 1 | 1 | 1 | 1 | 1 | 1 | 1 | 1 | 8 |
| Levolger | 2015 | 1 | 1 | 1 | 1 | 2 | 1 | 1 | 1 | 9 |
| Valero | 2015 | 1 | 1 | 1 | 1 | 2 | 1 | 1 | 1 | 9 |
| Voron | 2015 | 1 | 1 | 1 | 1 | 2 | 1 | 1 | 1 | 9 |
| Zhou | 2015 | 1 | 1 | 1 | 1 | 2 | 1 | 1 | 1 | 9 |
| Higashi | 2016 | 1 | 1 | 1 | 1 | 2 | 1 | 1 | 1 | 9 |
| Itoh | 2016 | 1 | 1 | 1 | 1 | 2 | 1 | 1 | 1 | 9 |
| Kamachi | 2016 | 1 | 1 | 1 | 1 | 2 | 1 | 1 | 1 | 9 |
| Takagi | 2016 | 1 | 1 | 1 | 1 | 2 | 1 | 1 | 1 | 9 |
| Yabusaki | 2016 | 1 | 1 | 1 | 1 | 2 | 1 | 0 | 1 | 8 |
| Begini | 2017 | 1 | 1 | 1 | 1 | 2 | 1 | 1 | 1 | 9 |
| Hiraoka | 2017 | 1 | 1 | 1 | 1 | 1 | 1 | 1 | 1 | 8 |
| Nishikawa | 2017 | 1 | 1 | 1 | 1 | 1 | 1 | 1 | 1 | 8 |
| Okumura | 2017 | 1 | 1 | 1 | 1 | 2 | 1 | 1 | 1 | 9 |
| Yuri | 2017 | 1 | 1 | 1 | 1 | 1 | 1 | 1 | 1 | 8 |
| Antonelli | 2018 | 1 | 1 | 1 | 1 | 2 | 1 | 1 | 1 | 9 |
| Ha | 2018 | 1 | 1 | 1 | 1 | 2 | 1 | 1 | 1 | 9 |
| Kobayashi | 2018 | 1 | 1 | 1 | 1 | 1 | 1 | 1 | 1 | 8 |
| Saeki | 2018 | 1 | 1 | 1 | 1 | 2 | 1 | 1 | 1 | 9 |
| Shiba | 2018 | 1 | 1 | 1 | 1 | 1 | 1 | 1 | 1 | 8 |
| Shirai | 2018 | 1 | 1 | 1 | 1 | 1 | 1 | 1 | 1 | 8 |
| Fujita | 2019 | 1 | 1 | 1 | 1 | 1 | 1 | 1 | 1 | 8 |
| Hamaguchi | 2019 | 1 | 1 | 1 | 1 | 2 | 1 | 1 | 1 | 9 |
| Imai | 2019 | 1 | 1 | 1 | 1 | 2 | 1 | 1 | 1 | 9 |
| Kobayashi | 2019 | 1 | 1 | 1 | 1 | 1 | 1 | 1 | 1 | 8 |
| Kroh | 2019 | 1 | 1 | 1 | 1 | 1 | 1 | 1 | 1 | 8 |
| Labeur | 2019 | 1 | 1 | 1 | 1 | 1 | 1 | 1 | 1 | 8 |
| Lee | 2019 | 1 | 1 | 1 | 1 | 2 | 1 | 1 | 1 | 9 |
| Yugawa | 2019 | 1 | 1 | 1 | 1 | 2 | 1 | 1 | 1 | 9 |
| Akce | 2020 | 1 | 1 | 1 | 1 | 1 | 1 | 1 | 1 | 8 |
| Bekki | 2020 | 1 | 1 | 1 | 1 | 1 | 1 | 1 | 1 | 8 |
| Choi | 2020 | 1 | 1 | 1 | 1 | 2 | 1 | 1 | 1 | 9 |
| Ebadi | 2020 | 1 | 1 | 1 | 1 | 1 | 1 | 1 | 1 | 8 |
| Endo | 2020 | 1 | 1 | 1 | 1 | 1 | 1 | 1 | 1 | 8 |
| Faron | 2020 | 1 | 1 | 1 | 1 | 2 | 1 | 1 | 1 | 9 |
| Kotoh | 2020 | 1 | 1 | 1 | 1 | 2 | 1 | 1 | 1 | 9 |
| Lanza | 2020 | 1 | 1 | 1 | 1 | 1 | 1 | 1 | 1 | 8 |
| Santhakuma | 2020 | 1 | 1 | 1 | 1 | 2 | 1 | 1 | 1 | 9 |
| Uojima | 2020 | 1 | 1 | 1 | 1 | 2 | 1 | 1 | 1 | 9 |
| Wu | 2020 | 1 | 1 | 1 | 1 | 2 | 1 | 0 | 0 | 7 |
| Yeh | 2020 | 1 | 1 | 1 | 1 | 2 | 1 | 1 | 1 | 9 |
| Deng | 2021 | 1 | 1 | 1 | 1 | 2 | 1 | 1 | 1 | 9 |
| Guichet | 2021 | 1 | 1 | 1 | 1 | 2 | 1 | 1 | 1 | 9 |
| Jang | 2021 | 1 | 1 | 1 | 1 | 2 | 1 | 1 | 1 | 9 |
| Li | 2021 | 1 | 1 | 1 | 1 | 1 | 1 | 1 | 1 | 8 |
| Liao | 2021 | 1 | 1 | 1 | 1 | 2 | 1 | 1 | 1 | 9 |
| Saeki | 2021 | 1 | 1 | 1 | 1 | 2 | 1 | 1 | 1 | 9 |
| Salman | 2021 | 1 | 1 | 1 | 1 | 1 | 1 | 0 | 1 | 7 |
| Yoshio | 2021 | 1 | 1 | 1 | 1 | 1 | 1 | 1 | 1 | 8 |
| Chien | 2022 | 1 | 1 | 1 | 1 | 1 | 1 | 1 | 1 | 8 |
| Dong | 2022 | 1 | 1 | 1 | 1 | 2 | 1 | 0 | 1 | 8 |
| Fujita | 2022 | 1 | 1 | 1 | 1 | 1 | 1 | 1 | 1 | 8 |
| Hayashi | 2022 | 1 | 1 | 1 | 1 | 2 | 1 | 1 | 1 | 9 |
| Hou | 2022 | 1 | 1 | 1 | 1 | 2 | 1 | 1 | 1 | 9 |
| Kim | 2022 | 1 | 1 | 1 | 1 | 2 | 1 | 1 | 1 | 9 |
| Roth | 2022 | 1 | 1 | 1 | 1 | 2 | 1 | 1 | 1 | 9 |
| Tamai | 2022 | 1 | 1 | 1 | 1 | 2 | 1 | 1 | 1 | 9 |
| Xiao | 2022 | 1 | 1 | 1 | 1 | 2 | 1 | 1 | 1 | 9 |
| Yang | 2022 | 1 | 1 | 1 | 1 | 2 | 1 | 1 | 1 | 9 |
| Zhang | 2022 | 1 | 1 | 1 | 1 | 2 | 1 | 1 | 1 | 9 |

**Table S3.** Subgroup analysis for overall survival

|  | **No. of Studies** | **HR** | **95% Confidence Interval** | ***P*-Value** | | **Cochran-Q** | ***I*^2** |
| --- | --- | --- | --- | --- | --- | --- | --- |
| **By ethnicity** |  |  |  | |  |  |  |
| Asian | 34 | 2.098 | 1.841-2.390 | | <0.001 | 0.002 | 46.3% |
| non-Asian | 13 | 2.177 | 1.781-2.663 | | <0.001 | 0.205 | 23.7% |
| **By type of liver cancer** |  |  |  | |  |  |  |
| HCC | 40 | 2.069 | 1.841-2.326 | | <0.001 | 0.005 | 40.8% |
| ICC | 4 | 2.912 | 2.150-3.943 | | <0.001 | 0.942 | 0.00% |
| **By treatment modalities** |  |  |  | |  |  |  |
| Curative | 23 | 2.454 | 2.010-2.996 | | <0.001 | 0.001 | 54.2% |
| Palliative | 20 | 1.932 | 1.710-2.184 | | <0.001 | 0.314 | 11.3% |
| **By sarcopenia definitions** |  |  |  | |  |  |  |
| L3-SMI | 32 | 2.069 | 1.825-2.347 | | <0.001 | 0.013 | 39.3% |
| L3-PMI | 9 | 2.361 | 1.684-3.311 | | <0.001 | 0.002 | 67.2% |
| FFMA(MRI) | 2 | 2.229 | 1.359-3.654 | | 0.001 | 0.526 | 0.0% |
| **By etiology of liver cancer** |  |  |  | |  |  |  |
| HCV | 2 | 5.283 | 2.141-13.036 | | <0.001 | 0.235 | 36.6% |
| Others | 45 | 2.061 | 1.854-2.290 | | <0.001 | 0.009 | 29.0% |

**Table S4.** Subgroup analysis for RFS/DFS

|  | **No. of Studies** | **HR** | **95% Confidence Interval** | ***P*-Value** | **Cochran-Q** | ***I*^2** |
| --- | --- | --- | --- | --- | --- | --- |
| **By ethnicity** |  |  |  |  |  |  |
| Asian | 9 | 1.649 | 1.42-1.911 | <0.001 | 0.486 | 0% |
| non-Asian | 2 | 2.252 | 1.350-3.756 | 0.002 | 0.173 | 46.20% |
| **By type of liver cancer** |  |  |  |  |  |  |
| HCC | 6 | 1.643 | 1.350-2.000 | <0.001 | 0.287 | 19.40% |
| ICC | 4 | 2.171 | 1.620-2.909 | <0.001 | 0.271 | 17.30% |
| **By sarcopenia definitions** | |  |  |  |  |  |
| L3-SMI | 8 | 1.657 | 1.415-1.939 | <0.001 | 0.448 | 0.0% |
| L3-PMI | 2 | 1.959 | 1.171-3.277 | 0.01 | 0.094 | 64.30% |
